# Development of a brief learning environment measure for use in healthcare professions education: the Healthcare Education Micro Learning Environment Measure (HEMLEM)

**DOI:** 10.1186/s12909-020-01996-8

**Published:** 2020-04-09

**Authors:** R. Isba, C. Rousseva, K. Woolf, L. Byrne-Davis

**Affiliations:** 1 Lancaster Medical School, Lancaster University, Bailrigg, Lancaster LA1 4YW England; 2 Emergency Department, North Manchester General Hospital, Delaunays Road, Crumpsall, Manchester, M8 5RB England; 3 Royal Liverpool Hospital, Prescot Street, Liverpool, L7 8XP England; 4 Research Department of Medical Education, UCL Medical School, Gower Street, London, WC1E 6BT England; 5 Division of Medical Education, University of Manchester, Stopford Building, Oxford Road, Manchester, M13 9PT UK

## Abstract

**Background:**

The learning environment impacts many aspects of healthcare education, including student outcomes. Rather than being a single and fixed phenomenon, it is made up of multiple micro learning environments. The standard clinical learning environment measurement tools do not consider such diversity and may fail to adequately capture micro learning environments.

Moreover, the existing tools are often long and may take a prohibitive amount of time to complete properly. This may have a negative impact on their usefulness in educational improvement strategies. In addition, there is no universal tool available which could be utilised across several healthcare student groups and placement settings.

**Aim:**

To create an evidence-based measurement tool for assessing clinical micro learning environments across several healthcare profession student groups.

**Methods:**

The measurement tool was developed through a step-wise approach: 1) literature review with iterative analysis of existing tools; 2) generation of new items via thematic analysis of student experiences; 3) a Delphi process involving healthcare educators; 4) piloting of the prototype; and 5) item reduction.

**Results:**

The literature review and experiential data from healthcare students resulted in 115 and 43 items respectively. These items were refined, leaving 75 items for the Delphi process, which produced a prototype with 57 items. This prototype was then completed by 257 students across the range of healthcare professions, with item reduction resulting in a 12-item tool.

**Conclusion:**

This paper describes a mixed methods approach to developing a brief micro learning environment measurement tool. The generated tool can be used for measuring student perceptions of clinical environments across several healthcare professions. Further cross-cultural and cross-professional validation studies are needed to support widespread use, possibly through mobile application.

## Background

The learning environment in healthcare education is a manifestation of the curriculum as experienced by students [1] and influences healthcare professions students in a number of ways, including behaviour [2], satisfaction [3], and educational outcomes [4–6].

Whilst much of healthcare students’ professional development occurs on clinical placements, there may be gaps between what they should be learning and what they actually learn [7]. Understanding how the environment contributes to student learning can be challenging as learning environments are complex and influenced at many organisational levels e.g. departmental, institutional, and national [8]. Student perceptions of learning environments are also affected by myriad factors such as physical space and organisational culture, but also by a student’s background and social relationships, and the degree to which they feel included in clinical activities [9–13]. As such, while the learning environment is usually conceived of as a unitary static concept, in reality it is likely to be comprised of several small and dynamic ‘micro environments’ as experienced by the student, e.g. a single primary care clinic or a day on a hospital ward [14].

The educational importance of learning environments is reflected in the numerous tools developed to measure them [15]. Traditionally, tools measured institutional-level phenomena as well as student-level factors, and may not have picked up the important subtleties of micro environments. Existing tools are also mostly designed for specific groups of students e.g. nurses, with limited re-visiting of their validity and reliability when used with groups [16–19]. Additionally, completion may be time-consuming relative to the amount of time students have spent in the learning environment itself. It has been suggested that we need new tools that measure the small, dynamic, micro environments experienced by students on clinical placement [8].

Measuring students’ experiences of micro learning environments on placement is valuable for numerous reasons. It can give insight into possible explanations for the gap between what should be taught, what is taught, and what is learned. It may also provide information about student experiences in real time, while contributing information about the overall setting of the placement [12]. This in turn allows for educational improvements to be made for all students – regardless of professional group – as part of continuous quality improvement [1]. Finally, the act of asking for students’ feedback on the learning environment can in itself contribute to a more positive environment, by showing students that their opinions are valued [8]. This engagement may also encourage students to exert more influence on their own learning environments, and those of the students around them [14].

Whilst a relatively large number of tools exist to measure learning environments in undergraduate and postgraduate healthcare professions education, these measures are unsuitable for the assessment of micro learning environments experienced by all healthcare professions students for two key reasons. Firstly, some are relatively long e.g. the Dundee Ready Educational Environment Measure, DREEM [20] has 50 items and the Clinical Learning Environment Inventory, CLEI [21] more than 40, and therefore may lack utility for a short (micro) placement. Secondly, none are designed to assess the experiences of all learners - they focus on individual professional groups. Although different groups of learners within a learning environment may have a number of profession-specific elements that need to be taken into account for successful learning to occur, the learning environment itself has some fundamental aspects experienced by all students. There is a need, therefore, for a brief instrument that can be used to assess the same learning environment as experienced by different groups of healthcare students.

The aim of this study was to create an evidence-based micro learning environment measure to provide information about placements in which students may have spent only a short period of time. Further, by reviewing existing literature and engaging students and educators across all healthcare groups, the intention was to develop a generic tool (the Healthcare Education Micro Learning Environment Measure – HEMLEM).

## Methods

### Item generation

#### Literature search and thematic analysis

A literature search was conducted including papers from January 1st 2005 to June 2015 in English. Databases searched were MEDLINE, HMIC, and CINAHL, plus the grey literature (e.g. health professions education conference abstracts) using the terms “learning OR educational” AND “environment OR climate” AND “measure” AND “health* OR med* OR nurs* OR dent* OR pharm* OR allied OR professional”. Ancestor searches were performed on the reference lists of key papers. Items from existing published scales were then extracted from the literature.

The literature was also analysed thematically. Codes describing elements of learning environments were created and grouped into themes, which were then converted into items. Duplicates or near duplicates of existing items in published scales were then removed.

### Qualitative examples of positive and negative learning environments

In November 2016, between 10,000 and 15,000 healthcare students from across the North West were invited to participate via email via Health Education England North West and relevant universities.. Data collection occurred via a web-based platform (the University of Manchester’s eForms) upon which students indicated their professional group (only – no further details were collected) and wrote free-text answers to the following:

* "Please describe an example of a positive learning environment that you have experienced (please do not include any identifiable information).
* Please describe an example of a negative learning environment that you have experienced (please do not include any identifiable information)."

It was not technically possible to ensure that a single participant did not submit multiple responses via the platform (as no identifiable information was collected, to ensure anonymity), so an assumption was made that all responses were from separate individuals. Qualitative data were independently thematically analysed by RI and CR, from which another list of items was created and combined with the previously identified and created items. New items (derived from the qualitative study involving a wide range of professional groups) that overlapped with or duplicated previous items (derived from the literature search and representing a smaller number of professional groups) were prioritised for inclusion in the prototype.

### Item reduction

#### Delphi process

The Delphi process aimed to gain expert consensus on the items important for inclusion [22–24]. Individuals from a variety of groups involved in healthcare education were invited to participate. The process was conducted online. Participants were presented with items and asked to indicate whether or not it gave crucial information (‘Yes - this is a crucial part of a learning environment’, ‘No’, and ‘Don’t know’), and to rate their confidence for a ‘Yes’ or ‘No’ response (from 1 - not at all confident, to 7 - extremely confident) or give free text comment for a ‘Don’t know’ response.

Items were retained if 75% of participants agreed it was crucial. Items were rejected if 75% of participants agreed it was not crucial. For those marginally below the 75% agreement cut-off, RI decided whether to retain based on the item relevance, clarity, and specificity, plus free text comments. After a single Delphi round, the items had only been reduced by around a quarter, so a decision was made to move directly to prototype pilot and item reduction.

#### Prototype pilot

All healthcare students in North West England were invited via email to complete an online pilot version of the prototype questionnaire in February and March 2017. Participants were asked to think about a current or recent clinical placement and indicate their agreement/disagreement with the 57 prototype items. Response options ranged from 1 - strongly disagree to 5 - strongly agree. They were also asked to indicate their professional group.

#### Psychometric item reduction

The items were analysed statistically to understand whether the questionnaire was measuring one or more aspects of the learning environment (“constructs”) and also to enable the removal of items that did not appear to be measuring what was intended, and thus create a shorter version of the questionnaire. The reduction process followed Streiner and Norman [25], and Goetz et al. [26]. All items were assessed for quality using *p* values (proportion correct), skewedness and kurtosis statistics (the distribution of the scores), inter-item correlations (the statistical relationship between the scores on each item), item-total correlations (the statistical relationship between each item and the overall score), and Cronbach’s alpha (the internal consistency or reliability of the questionnaire as a whole). Items with mean inter-item correlations of at least 0.25 were included even if highly skewed, and items with a *p* value of over 0.95 or under 0.05, or with an item-total correlation below 0.20 were excluded. After excluding poor quality items, the dimensionality of the prototype version of the questionnaire (i.e. whether the questionnaire was measuring one or more underlying aspects of the learning environment) was examined using exploratory factor analysis, with scree plots and factor loadings being used to establish the factor structure. Items with the highest loadings on each factor identified (i.e. the items that had the closest statistical relationship with a particular factor) were examined for quality using item statistics and for meaning using item wording. Of those, 10–15 items with the highest quality scores and that appeared qualitatively to measure different aspects of each factor identified were chosen for inclusion in the short questionnaire.

Total and mean scores for the short questionnaire and for each of the short subscales were calculated by summing and averaging items scores, respectively. Cronbach’s alpha was used to calculate the reliability of the short version and the reliability of the subscales. The similarity of scores on the prototype and short versions was assessed using Pearson correlations and examination of means and standard deviations.

## Results

### Item generation

**Fig. 1:**
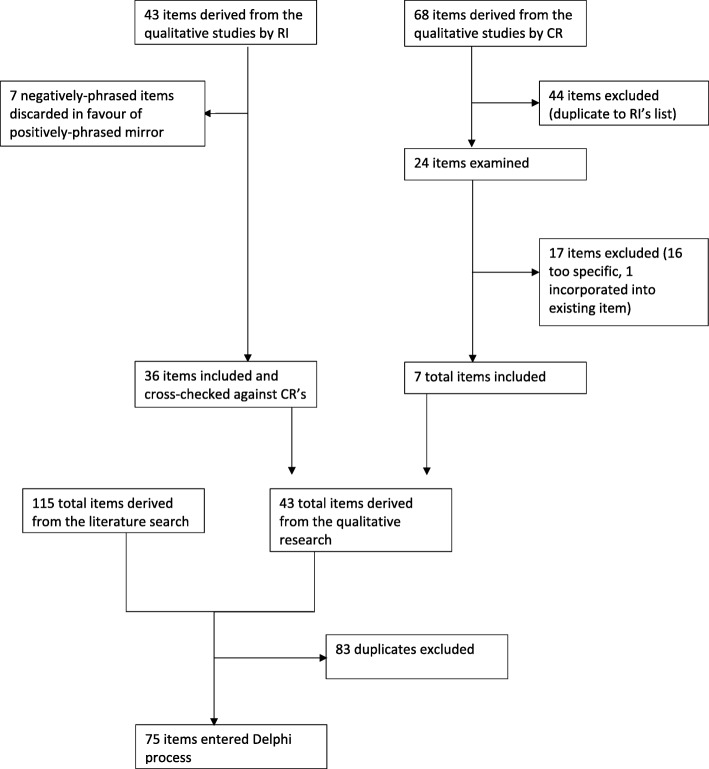
Overview of production of items for the Delphi process

Analysis of existing scales yielded 394 items and 288 statements, grouped into 12 themes, were identified from the literature. After removal of duplicates, there were 115 items from existing published scales, which also covered all relevant themes from the literature (Fig. 1).

Seventy-five respondents gave consent to provide examples of positive and negative experiences, but six did not provide any other data, resulting in data from 69 participants. Twenty-nine were nursing students, 11 were physiotherapy students, and the remaining respondents were from medicine (*n* = 1), midwifery (*n* = 4), occupational therapy (*n* = 8), orthoptics (n = 4), podiatry (*n* = 2), radiography (n = 8), and prosthetics/orthotics (*n* = 2).

From the responses, RI and CR created a total of 131 items (RI = 43, CR = 68). Using RI’s items as a base, seven negatively-phrased items were removed in favour of their positively-phrased mirror counterparts, leaving 36 items. These items were then cross-checked against CR’s list, identifying 24 items that were not already covered. Of these, one was incorporated into an existing item, 16 were discarded as too specific/fine detail, and seven were added to the list of items for consideration.

These 43 items from the qualitative work were then combined with the 115 derived from the literature (Fig. 1). Deletion of duplicates/near duplicates gave a final set of 75 items for inclusion in the Delphi process. Of these, one was an original item from an existing scale, three were combined from new and existing items, 31 were adapted from existing items, and 40 derived from the new data (Supplementary Table A).

### Delphi process

Twenty-eight people took part in the Delphi process, representing eight professional groups (nurse = 15; physiotherapist = 8; doctor = 3; occupational therapist = 1; practice education facilitator = 1) and managing placements across primary care (9/28), secondary care (20/28), and universities s (9/28) (participants could manage placements in more than one place of work). Fifty-four of the 75 items (72%) reached the consensus threshold for inclusion. An additional three items were included because they reached near consensus, addressed an important issue, or represented something not included elsewhere. These 57 items were included in the prototype.

### Piloting of prototype instrument

Two hundred and fifty-seven healthcare students from 16 professional groups completed the 57-item prototype. Adult nursing students were the biggest group (*n* = 88), with nursing students of all types making up just over half of all respondents (*n* = 139; 54.1%). Other professional groups represented were: midwife (*n* = 39), radiographer (*n* = 17), occupational therapist (*n* = 16), doctor (*n* = 14), operating department practitioner (*n* = 12), physiotherapist (*n* = 9), assistant practitioner (*n* = 4), podiatrist (*n* = 3), audiologist (*n* = 1), health visitor (*n* = 1), orthoptist (*n* = 1), and cardiac rehabilitation practitioner (*n* = 1).

### Factor analysis

**Fig. 2:**
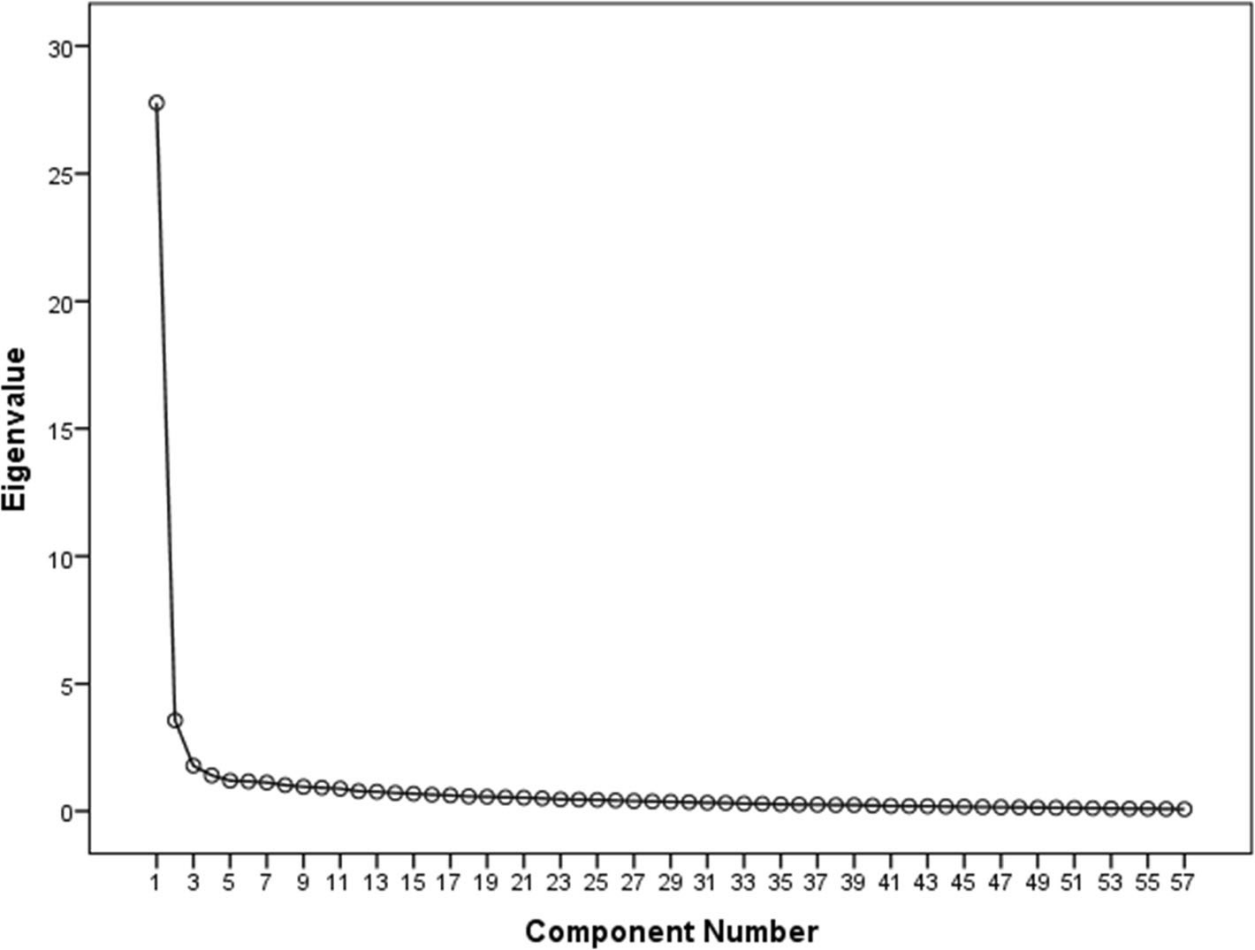
Scree plot for the first exploratory principal components analysis

Items were generally negatively skewed, meaning students tended to give high (positive) scores. Cronbach’s alpha for the 57-item version was 0.98. The mean inter-item correlation was 0.45 and the mean item-total correlation was 0.67, with no items excluded from the factor analysis based on a priori quality criteria. All 57 items were entered into a principal components analysis with a Varimax rotation and Kaiser normalisation. The principal components analysis was an exploratory exercise to understand how many underlying factors the questionnaire was measuring. The results showed eight factors were extracted, however, visualisation of the results using a scree plot suggested there were in fact two or three factors. We therefore explore the two-factor and three-factor solutions to see which were a better fit to the data (Fig. 2).

First, we looked at how much of the variance in results was explained by each factor. In the three-factor solution the first factor explained 24% of variance, the first two together explained 40% and three factors together explained 50%. In the two-factor solution, the first factor explained 31% of the variance and both factors together explained 55% of the variance. This suggested that the two-factor solution was a better fit. Table B (Supplementary materials) shows the factor loadings for the two-factor solution. The two factors are described below:

*Factor 1 (Staff attitudes and behaviours)* related to student perceptions of staff attitudes and behaviours, including how friendly and welcoming staff were, how much they valued student input, were supportive, and cared about students and teaching. 24 items had loadings of > 0.6 on to this factor.

*Factor 2 (Teaching quality)* related to perceptions of teaching quality, including whether teaching provided opportunities to develop knowledge and skills, was patient focussed, and was tailored to student needs. 13 items had loadings of > 0.6 on this factor.

### Item reduction

All items were of sufficient quality for inclusion in the short HEMLEM, except item 55, which had had high skewedness, high kurtosis, and low item-total correlation (Table 1). Items in subscale 1 ‘Staff attitudes and behaviours’ cover perceptions that staff are supportive, value student input, and are interested in students and teaching. Items in subscale 2 ‘Teaching quality’ cover the development of knowledge and skills, teaching being tailored to student needs, and teaching being patient-focused.

**Table 1:** Description of items included in final HEMLEM

	Item number and wording	Factor loading	Skewedness	Corrected Item-Total Correlation	Source
Subscale 1: Staff attitudes and behaviours	1 This placement had a welcoming, friendly, and open atmosphere.	.833	−.902	.824	New from data
	2 There was a culture where I felt free to ask questions or make comments on this placement.	.817	−1.173	.817	Adapted MCTQ [27]
	3 Staff on this placement were enthusiastic about teaching.	.750	−.842	.782	New from data
	4 My supervisor showed an interest in me.	.680	−.975	.757	Adapted MCTQ [27]
	5 My input was valued on this placement.	.658	−.853	.782	New from data
	6 I was provided with regular, useful, and supportive feedback during this placement.	.651	−.780	.769	New from combined existing items and new data
Subscale 2: Teaching quality	7 I had the opportunity to apply my previous knowledge in this placement.	.783	−.975	.728	Adapted CLEQ [28]
	8 My knowledge and skills were developed on this placement.	.737	−1.142	.797	New from data
	9 This placement helped me put theory into practice.	.677	−.981	.683	New from data
	10 I was able to meet my learning objectives on this placement.	.668	−1.129	.714	New from data
	11 I had the opportunity to deal with the patient as a whole on this placement.	.657	−.798	.608	Adapted CLEQ [28]
	12 I was given tasks suitable for my stage of training on this placement.	.649	−1.110	.713	New from data

The reliability (Cronbach’s alpha) of subscale 1 was 0.93; of subscale 2 was 0.87; and of the 12-item scale was 0.93. The 12 chosen items were entered into a confirmatory factor analysis with a Varimax rotation. Together both factors explained 68% of the variance in scores.

### Psychometrics of the HEMLEM and comparison with 57-item prototype

Mean scores for the 12-item HEMLEM and the two 6-item subscales were negatively skewed, reflecting the skewing of items in the longer prototype. The mean score on Subscale 1 (Staff attitudes and behaviours) was 3.97 (SD 0.96); the mean score on Subscale 2 (Teaching quality) was 4.13 (SD 0.77). The subscales were also significantly and highly correlated (*r* = 0.676; *p* < .001). The 57-item and 12-item versions were similar in terms of distributions, mean scores and standard deviations (Supplementary data). They were also highly statistically significantly correlated (*r* = 0.966; *p* < .001).

## Discussion

**Fig. 3:**
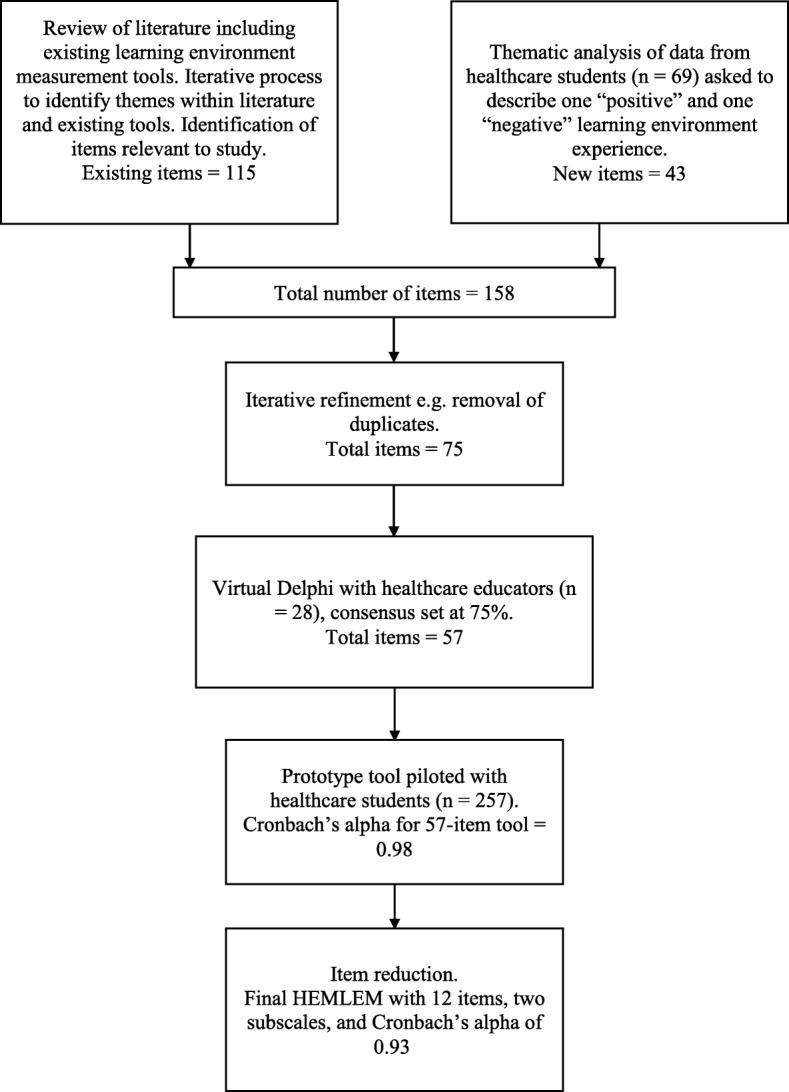
Study flow chart

The 12-item HEMLEM scale to measure students’ perceptions of the quality of a clinical micro learning environment was created by a literature review, qualitative analysis of written student examples, a Delphi process with educators, prototype testing with students, and psychometric item reduction (Fig. 3 for overview of study). All final HEMLEM items are phrased positively and scored from 1 (strongly disagree) to 5 (strongly agree). There are 12 items, therefore the total score for HEMLEM can be presented as out of a possible 60. Two subscales, each with six items, were identified using factor analysis. The subscales had very high reliability and could thus be used individually (although it is recommended that both are used together).

All of the participants in this study were drawn from a single region within England, which presents a weakness that means further testing is needed to assess the HEMLEM’s transferability to other countries and settings. More extensive piloting of the tool could explore whether the tool can identify quality assurance issues, as people tend to rate their environments highly, resulting in the negatively skewed data shown here.

The response rate (in the low single figures throughout) means that those who responded may be different to the rest of the student population from which they were drawn, which is potentially a major weakness. When using the online platform we prioritised anonymity and this may have resulted in individual participants entering multiple responses, which may in turn have influenced the results, and is therefore a potential limitation.

Those who participated in the prototype study had very good experiences, so we can assume that we did not have representation of the full range of student experiences at that stage (although we had asked about both positive and negative experiences in the pre-Delphi). The concern with having a small range of participant experiences is that correlations (e.g. between items) tend to be lower [27], and this could in theory impact on the results of the factor analysis. However, in this case the correlations were very high, so perhaps the low response rate and possibly limited range of experiences did not have an appreciable impact on the data and outcomes of interest in this study. However, the low response rates (which we were unable to accurately calculate) remain a potential major weakness, even in the context of our findings, although given the expansion in recruitment methods for studies of this kind e.g. via social media, response rates may not always be calculable [28].

It is interesting that the meta-synthesis and the primary data collection did not include variables outlining how the students themselves contribute to the learning environment. This might be a limitation of the study design, in which students were asked to reflect on their learning environments and might not recognise the bi-directional nature of the relationship between student and learning environment. Future inclusion of a consideration of this bi-directionality would fit well with recent models for increasing student engagement [29, 30].

A particular strength is that this study used mixed methodology to create HEMLEM. The final instrument is quick and easy to use, and can be used to gather data from a variety of different student groups within the same learning environment. The HEMLEM is also designed to enable the assessment of learning environments even when they are experienced only for a relatively short amount of time e.g. a single day.

The 12-item scale should now be validated on a new sample of healthcare students from different professional groups and in different locations internationally. It may also be possible to create a smartphone app based on HEMLEM that students could give real-time feedback on placements, similar to Form^2^, an ipad-native tool used at the University of Manchester to collect other feedback.

## Conclusion

This paper describes a mixed methods approach to developing a brief micro learning environment measure that is psychometrically robust and evidence-based, and that can be used to assess the experience of any student, from any healthcare professions group, on any clinical placement, no matter how brief in duration.

### Practice points

- There was good consensus between literature, students and educators, about the features that make good and poor learning environments.
- It is possible to capture the consensus features of a good micro learning environment in a 12-item questionnaire, completed by the learner.
- Two factors are important for a good micro learning environment: teaching quality and staff attitudes and behaviours.

## Supplementary information

**Additional file 1: Table A.** Items for inclusion in Delphi including origins (items marked in bold are negatively phrased) [31–36]. **Table B:** Varimax rotated component matrix for the two factor solution. * Item loaded > = 0.6 onto factor 1; ** item loaded > = 0.6 onto factor 2; ^ item loaded equally (within 0.1) onto both factors. Loadings smaller or equal to 0.2 not shown. **Figure A.** Distribution of mean scores for the 57-item long scale, for the 12-item short scale, and for subscales 1 and 2.

## Data Availability

The datasets used and/or analysed during the current study are available from the corresponding author on reasonable request.

## Abbreviations

CLEI: Clinical Learning Environment Inventory
CLEQ: Clinical Learning Evaluation Questionnaire
CR: Dr. Christiana Rousseva
DRECT: Dutch Residency Educational Climate Test
DREEM: Dundee Ready Educational Environment Measure
HEMLEM: Health Education Micro Learning Environment Measure
KW: Dr. Kath Woolf
LBD: Dr. Lucie Byrne-Davis
MCPI: Manchester Clinical Placement Index
MCTQ: Maastricht Clinical Teaching Questionnaire
PHEEM: Postgraduate Hospital Educational Environment Measure
PVTEM: Practice Vocational Training Environment Measure
RI: Professor Rachel Isba
SD: Standard deviation
