# Supplementary material for: Development of a brief learning environment measure for use in healthcare professions education: the Healthcare Education Micro Learning Environment Measure (HEMLEM)
Source: BMC Med Educ. 2020 Apr 9;20:110. doi: 10.1186/s12909-020-01996-8 (PMC7146917; doi:10.1186/s12909-020-01996-8)
Supplement: Supplementary file 1 — Additional file 1: Table A. Items for inclusion in Delphi including origins (items marked in bold are negatively phrased) [31–36]. Table B: Varimax rotated component matrix for the two factor solution. * Item loaded > = 0.6 onto factor 1; ** item loaded > = 0.6 onto factor 2; ^ item loaded equally (within 0.1) onto both factors. Loadings smaller or equal to 0.2 not shown. Figure A. Distribution of mean scores for the 57-item long scale, for the 12-item short scale, and for subscales 1 and 2. [file 12909_2020_1996_MOESM1_ESM.docx]

Supplementary material for HEMLEM

**Table A.** Items for inclusion in Delphi including origins (items marked in bold are negatively phrased).

| **Item (numbered for Delphi)** | **Source** | **Included in prototype** |
| --- | --- | --- |
| 1. I was expected on this placement. | New from data | * |
| 1. I was welcomed on this placement. | New from data | * |
| 1. I was able to prepare for this placement in advance. | New from data | * |
| 1. I experienced real-patient learning on this placement. | New from data | * |
| 1. I was supported to learn from any mistakes on this placement. | New from data |  |
| 1. Staff on this placement were enthusiastic about teaching. | New from data | * |
| 1. Staff seemed pleased to have students working with them. | New from data | * |
| 1. Staff were friendly and approachable. | New from data | * |
| 1. This placement helped me put theory into practice. | New from data | * |
| 1. I was able to access facilities and learning materials on this placement. | New from data | * |
| 1. I felt physically comfortable on this placement. | New from data |  |
| 1. This placement was a good learning experience. | New from data | * |
| 1. This placement offered me the opportunity to learn something new. | New from data |  |
| 1. This placement involved hands-on experience. | New from data | * |
| 1. I have learned from other healthcare professionals on this placement. | New from data | * |
| 1. Other students on this placement have been supportive. | New from data |  |
| 1. I have been supported and encouraged on this placement. | New from data | * |
| 1. I was provided with regular, useful, and supportive feedback during this placement. | New from combined data and existing items | * |
| 1. I didn’t feel like a burden on this placement. | New from data |  |
| 1. This placement had a welcoming, friendly, and open atmosphere. | New from data | * |
| 1. There was a culture where I felt free to ask questions or make comments on this placement. | Adapted MCTQ^27^ | * |
| 1. I have been able to learn from patients on this placement. | New from data | * |
| 1. I was given tasks suitable for my stage of training on this placement. | New from data | * |
| 1. I have seen some examples of good communication on this placement. | New from data | * |
| 1. I have gained confidence on this placement. | New from data | * |
| 1. My input was valued on this placement. | New from data | * |
| 1. I felt I made a valuable contribution on this placement. | New from data |  |
| 1. I was able to be an active part of the team on this placement. | New from data | * |
| 1. **I was just used as an extra set of hands on this placement.** | New from data |  |
| 1. **This placement was short-staffed.** | New from data |  |
| 1. The number of students on this placement was appropriate. | Adapted CLEQ^28^ | * |
| 1. **I was embarrassed, humiliated, or undermined on this placement.** | New from data | * |
| 1. I was given the right amount of responsibility on this placement. | New from data | * |
| 1. **Staff didn’t have the time to teach on this placement.** | New from data |  |
| 1. I was able to meet my learning objectives on this placement. | New from data | * |
| 1. **I felt like I was in the way on this placement.** | New from data |  |
| 1. My knowledge and skills were developed on this placement. | New from data | * |
| 1. I was able to identify a positive role model on this placement. | New from data | * |
| 1. I was given adequate supervision on this placement. | New from data | * |
| 1. I was able to learn at my own pace on this placement. | New from data |  |
| 1. **Staff on this placement appeared to have low morale.** | New from data |  |
| 1. **I worried that patients were receiving unsafe care on this placement.** | New from data | * |
| 1. **There was a mismatch between what I had been taught to do and what I was expected to do on this placement.** | New from data |  |
| 1. I had the opportunity to deal with the patient as a whole on this placement. | Adapted CLEQ^28^ | * |
| 1. I had the opportunity to apply my previous knowledge in this placement. | Adapted CLEQ^28^ | * |
| 1. I had the opportunity to apply a patient-centred approach on this placement. | Adapted CLEQ^28^ | * |
| 1. My workload on this placement was okay. | Adapted PHEEM^29^ |  |
| 1. People took time to listen to me on this placement. | Adapted CLEI^30^ | * |
| 1. I had the opportunity to communicate with patients and their families on this placement. | Adapted CLEQ^28^ | * |
| 1. There was a no-blame culture in this placement. | Adapted PHEEM^29^ | * |
| 1. There was a culture of mutual respect on this placement. | Adapted PHEEM^29^ | * |
| 1. As a student I was received in a positive way by patients. | Adapted PVTEM^31^ | * |
| 1. Differences of opinion on this placement did not have a negative impact. | Adapted DRECT^12^ |  |
| 1. As a student I was received in a positive way by staff. | Adapted UCEEM^13^ | * |
| 1. The atmosphere on this placement motivated me as a learner. | Adapted DREEM^20^ | * |
| 1. Students look forward to this placement. | Adapted CLEI^30^ |  |
| 1. After working on this placement, I felt a sense of satisfaction. | Adapted CLEI^30^ | * |
| 1. **This placement was a waste of time.** | Adapted CLEI^30^ |  |
| 1. I feel that this placement contributed to my professional development. | Adapted DREEM^20^ | * |
| 1. I had influence over my learning on this placement. | Adapted UCEEM^13^ | * |
| 1. There was visible leadership on this placement. | Adapted MCPI^32^ | * |
| 1. On this placement, educational activities took place as planned. | Adapted DRECT^12^ |  |
| 1. The teaching time on this placement was put to good use. | Adapted DREEM^20^ | * |
| 1. Staff went out of their way to help students on this placement. | Adapted CLEI^30^ | * |
| 1. My supervisor showed an interest in me. | Adapted MCTQ^27^ | * |
| 1. Staff were accessible to me on this placement. | Adapted PHEEM^29^ | * |
| 1. When I needed senior support I could always find someone. | Adapted DRECT^12^ | * |
| 1. The amount of supervision I received was appropriate for my level of experience | Adapted DRECT^12^ | * |
| 1. People took time to explain things to me when I asked for advice. | Adapted DRECT^12^ | * |
| 1. I was treated with respect on this placement. | Adapted DRECT^12^ | * |
| 1. This placement was student-centred. | Adapted DREEM^20^ | * |
| 1. Teamwork was an important part of this placement. | Adapted DRECT^12^ | * |
| 1. Everyone on this placement was treated equally regardless of gender, religion, or cultural background. | New combined from existing | * |
| 1. This placement stimulated me to think about how to improve my strengths and weaknesses. | New combined from existing | * |
| 1. This placement was appropriately organised. | Original MCPI^32^ | * |

**Table B:** Varimax rotated component matrix for the two factor solution.

* Item loaded >= 0.6 onto factor 1; ** item loaded >=0.6 onto factor 2; ^ item loaded equally (within 0.1) onto both factors. Loadings smaller or equal to 0.2 not shown.

| Item | | Factor | |
| --- | --- | --- | --- |
|  |  | 1 | 2 |
| 1 | I was expected on this placement. | .368 | .224 |
| 2 | I was welcomed on this placement.* | .742 | .238 |
| 3 | I was able to prepare for this placement in advance. | .236 | .397 |
| 4 | I experienced real-patient learning on this placement.** |  | .720 |
| 5 | Staff on this placement were enthusiastic about teaching.* | .725 | .385 |
| 6 | Staff seemed pleased to have students working with them.* | .733 | .311 |
| 7 | Staff were friendly and approachable.* | .817 | .222 |
| 8 | This placement helped me put theory into practice.** | .250 | .677 |
| 9 | I was able to access facilities and learning materials on this placement.^ | .418 | .475 |
| 10 | This placement was a good learning experience.** | .453 | .674 |
| 11 | This placement involved hands-on experience.** |  | .756 |
| 12 | I have learned from other healthcare professionals on this placement. | .322 | .506 |
| 13 | I have been supported and encouraged on this placement.* | .769 | .430 |
| 14 | I was provided with regular, useful, and supportive feedback during this placement.* | .651 | .453 |
| 15 | This placement had a welcoming, friendly, and open atmosphere.* | .833 | .258 |
| 16 | There was a culture where I felt free to ask questions or make comments on this placement.* | .817 | .287 |
| 17 | I have been able to learn from patients on this placement.** | .207 | .606 |
| 18 | I was given tasks suitable for my stage of training on this placement.** | .406 | .649 |
| 19 | I have seen some examples of good communication on this placement. | .581 | .447 |
| 20 | I have gained confidence on this placement.** | .452 | .643 |
| 21 | My input was valued on this placement.* | .658 | .468 |
| 22 | I was able to be an active part of the team on this placement.^ | .523 | .586 |
| 23 | The number of students on this placement was appropriate. | .478 | .206 |
| 24 | I was given the right amount of responsibility on this placement.^ | .453 | .568 |
| 25 | I was able to meet my learning objectives on this placement.** | .361 | .668 |
| 26 | My knowledge and skills were developed on this placement.** | .391 | .737 |
| 27 | I was able to identify a positive role model on this placement.^ | .495 | .588 |
| 28 | I was given adequate supervision on this placement.* | .707 | .370 |
| 29 | I had the opportunity to deal with the patient as a whole on this placement.** |  | .659 |
| 30 | I had the opportunity to apply my previous knowledge in this placement.** |  | .783 |
| 31 | I had the opportunity to apply a patient-centred approach on this placement. |  | .624 |
| 32 | People took time to listen to me on this placement.* | .656 | .459 |
| 33 | I had the opportunity to communicate with patients and their families on this placement. |  | .618 |
| 34 | There was a no-blame culture in this placement. | .532 | .204 |
| 35 | There was a culture of mutual respect on this placement.* | .729 | .270 |
| 36 | As a student I was received in a positive way by patients.^ | .289 | .341 |
| 37 | As a student I was received in a positive way by staff.* | .814 | .231 |
| 38 | The atmosphere on this placement motivated me as a learner.* | .701 | .433 |
| 39 | After working on this placement, I felt a sense of satisfaction.^ | .560 | .592 |
| 40 | I feel that this placement contributed to my professional development.** | .379 | .720 |
| 41 | I had influence over my learning on this placement. | .392 | .579 |
| 42 | There was visible leadership on this placement.^ | .463 | .431 |
| 43 | The teaching time on this placement was put to good use.^ | .583 | .542 |
| 44 | Staff went out of their way to help students on this placement.* | .722 | .355 |
| 45 | My supervisor showed an interest in me.* | .680 | .367 |
| 46 | Staff were accessible to me on this placement.* | .783 | .367 |
| 47 | When I needed senior support I could always find someone.* | .642 | .359 |
| 48 | The amount of supervision I received was appropriate for my level of experience* | .645 | .440 |
| 49 | People took time to explain things to me when I asked for advice.* | .723 | .327 |
| 50 | I was treated with respect on this placement.* | .822 | .237 |
| 51 | This placement was student-centred.* | .725 | .320 |
| 52 | Teamwork was an important part of this placement.* | .648 | .329 |
| 53 | Everyone on this placement was treated equally regardless of gender, religion, or cultural background.* | .632 |  |
| 54 | This placement stimulated me to think about how to improve my strengths and weaknesses.** | .305 | .630 |
| 55 | This placement was appropriately organised.^ | .543 | .521 |
| 56 | I was embarrassed, humiliated, or undermined on this placement. | .597 |  |
| 57 | I worried that patients were receiving unsafe care on this placement. | .424 |  |

**Figure A.** Distribution of mean scores for the 57-item long scale, for the 12-item short scale, and for subscales 1 and 2.
